# An Honor Worthily Bestowed

**Published:** 1873-05

**Authors:** 


*An Honor Worthily Bestowed.*

Dr. J. M. Toner, of Washington City, and one of the corresponding editors of the RECORD, was elected President of the American Medical Association, at its late meeting in St. Louis, Missouri. Dr. Toner is a splendid type of the American physician, and the honor bestowed upon him by the National Association will gratify hundreds of his friends in every portion of the country.

